# Electrodeposition of reduced graphene oxide with chitosan based on the coordination deposition method

**DOI:** 10.3762/bjnano.9.111

**Published:** 2018-04-17

**Authors:** Mingyang Liu, Yanjun Chen, Chaoran Qin, Zheng Zhang, Shuai Ma, Xiuru Cai, Xueqian Li, Yifeng Wang

**Affiliations:** 1 School of Materials Science and Engineering, Wuhan University of Technology, 122 Luoshi Road, Wuhan 430070, China

**Keywords:** chitosan, coordination, electrodeposition, nanocomposite films, reduced graphene oxide

## Abstract

The electrodeposition of graphene has drawn considerable attention due to its appealing applications for sensors, supercapacitors and lithium-ion batteries. However, there are still some limitations in the current electrodeposition methods for graphene. Here, we present a novel electrodeposition method for the direct deposition of reduced graphene oxide (rGO) with chitosan. In this method, a 2-hydroxypropyltrimethylammonium chloride-based chitosan-modified rGO material was prepared. This material disperses homogenously in the chitosan solution, forming a deposition solution with good dispersion stability. Subsequently, the modified rGO material was deposited on an electrode through codeposition with chitosan, based on the coordination deposition method. After electrodeposition, the homogeneous, deposited rGO/chitosan films can be generated on copper or silver electrodes or substrates. The electrodeposition method allows for the convenient and controlled creation of rGO/chitosan nanocomposite coatings and films of different shapes and thickness. It also introduces a new method of creating films, as they can be peeled completely from the electrodes. Moreover, this method allows for a rGO/chitosan film to be deposited directly onto an electrode, which can then be used for electrochemical detection.

## Introduction

Graphene has attracted tremendous attention due to its large surface area, excellent mechanical strength, high electronic conductivity and good adsorption capacity [1–2].Graphene has a diverse range of applications in solar cells, hydrogen storage materials, electroluminescent devices and electrode materials [3–5]. In particular, graphene or reduced graphene oxide (rGO) and biopolymer (e.g., gellan gum, chitosan, and alginate) nanocomposites have also gained growing interest in the development of advanced materials [6–8].

The electrodeposition technique has drawn extensive attention lately since it offers an effective convergent method to integrate biology with microelectronics to build the bio-device interface, which enables incredible potential in the area of lab-on-a-chip devices, biofuel cells and implantable devices [9]. Aminopolysaccharide chitosan is one of the most widely used materials for electrodeposition. Due to its pH-responsive film-forming properties, chitosan can be electrodeposited as a stable film on the cathode through a cathodic neutralization mechanism [10–12]. In addition to the cathodic electrodeposition of chitosan, our group has developed a novel electrodeposition method for chitosan, based on the coordination of chitosan with the metal ions [13]. The coordination electrodeposition of chitosan is promising for uses in surface coatings, sensors and metallic biomaterials, however, to date little attention has been paid to this coordination electrodeposition method.

A feature of the chitosan electrodeposition method is that it enables a controllable means to assemble nanoparticles (e.g., silver nanoparticles, carbon nanotubes, manganese oxides nanoparticles, and carbon dots) on electrodes through codeposition with chitosan, which offers attractive applications in antimicrobial coatings, biosensors, microbial fuel cells, and energy storage materials [14–18]. Among the studies on the codeposition of nanoparticles, some have been devoted to the electrodeposition of graphene with chitosan. Specifically, a few researchers have investigated the direct codeposition of graphene (or rGO) with chitosan on electrodes [19–21]. For instance, Qi et al. prepared rGO–chitosan hybrid films on an indium tin oxide (ITO) electrode through the codeposition of rGO and chitosan [21]. The main limitation of this method is that graphene disperses poorly and has poor colloidal stability in most common solvents [22–23]. In our previous experiments, it was found that the unmodified rGO was very difficult to disperse in the chitosan solution to form a stable deposition solution, which extremely hindered its further electrodeposition. For this reason, many researchers employ graphene oxide, which disperses well in the chitosan solution to carry out the electrodeposition of graphene. For example, Yang et al. reported that after electrodeposition in the graphene oxide/chitosan solution, graphene nanosheets could be electrodeposited onto the glassy carbon electrode through the electrochemical reduction of graphene oxide [24]. However, it has been reported that the electrochemically reduced graphene oxide contains a high amount of oxygen-containing groups, suggesting only the partial reduction of graphene oxide during the electrodeposition [25–26]. This approach can generate hydrogen bubbles during the electrochemical reduction process, introducing defects which limit the subsequent applications of the deposited film [27–28].

As mentioned above, the appealing applications of graphene electrodeposition, as well as the main limitations in the current electrodeposition method, stimulate our further studies in this direction. The purpose of this study is to develop a novel electrodeposition method for rGO based on the coordination deposition. This method allows for the direct electrodeposition of rGO (not using graphene oxide) with chitosan to achieve homogeneous coatings on the electrodes. Using this method, we can conveniently build rGO/chitosan films on electrodes, which simplifies the creation of sensors for electrochemical detection. Moreover, the method enables a controllable and new means to fabricate rGO/chitosan films which can be detached from the electrodes and used independently.

## Results and Discussion

### Electrodeposition of 2-hydroxypropyltrimethylammonium chloride chitosan-modified rGO (HACC-rGO)

**Figure 1:**
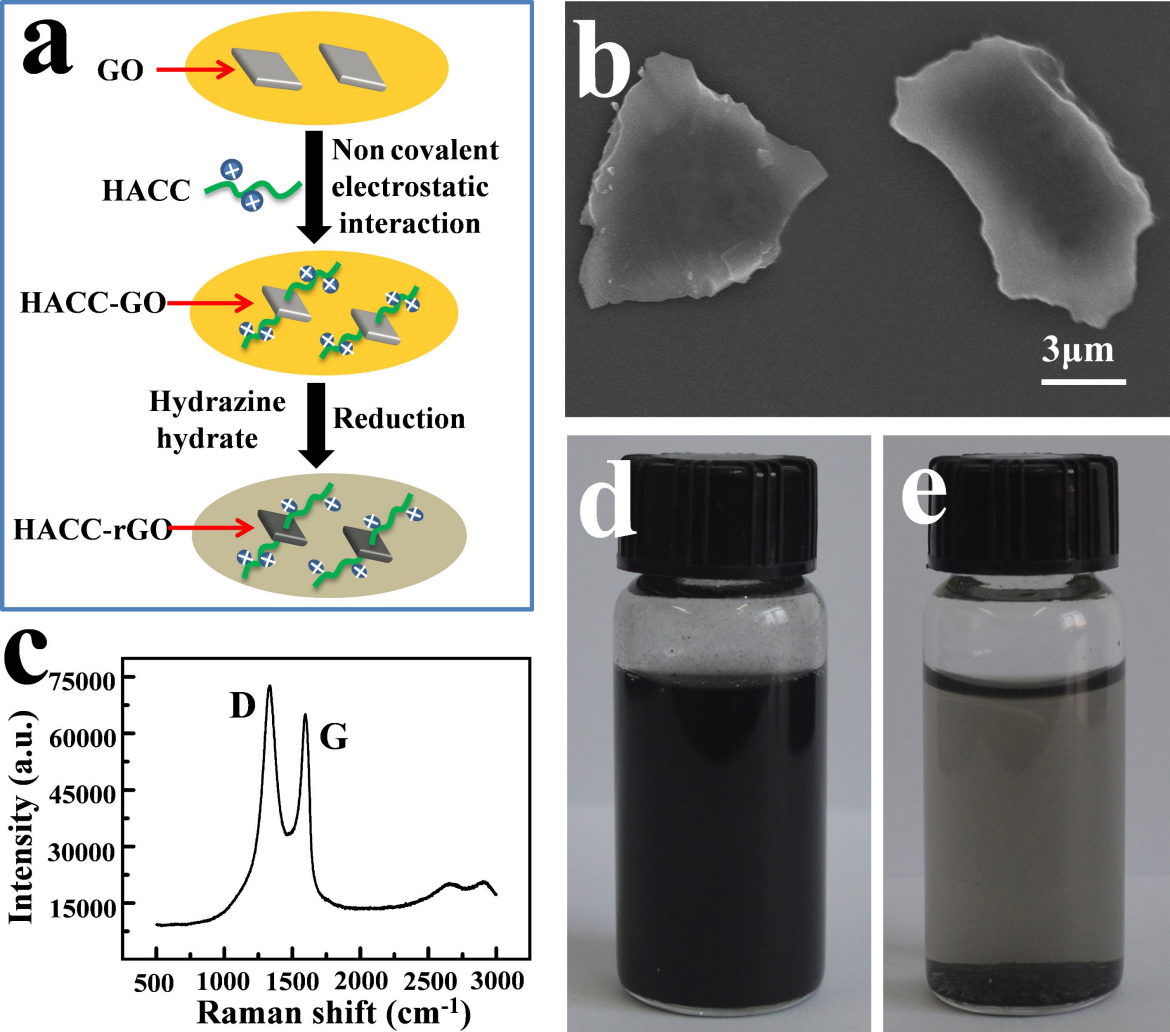
(a) Schematic of the preparation approach of HACC-rGO. (b) SEM image of the resulting HACC-rGO. (c) Raman spectrum of the HACC-rGO sample. (d) Photograph of the mixed solution of HACC-rGO and chitosan. (e) Photograph of the mixed solution of unmodified rGO and chitosan.

Graphene is attracting more attention due to its promising applications in many fields [3–5]. However, its application is limited due to the fact that it aggregates and disperses poorly in solvents, which seriously limits its application [22–23]. It was found that the unmodified rGO had poor dispersion in the chitosan solution, so it was difficult to form a stable deposition solution to perform the electrodeposition. To solve this problem, HACC-rGO was prepared according to our previously reported method [29]. The preparation approach of HACC-rGO is illustrated in Figure 1a. In this approach, the 2-hydroxypropyltrimethylammonium chloride chitosan-modified graphene oxide (HACC-GO) was prepared at first through the noncovalent electrostatic interaction between 2-hydroxypropyltrimethylammonium chloride chitosan (HACC) and graphene oxide (GO), and subsequently the HACC-GO was reduced by hydrazine hydrate to obtain the HACC-rGO. In our previous work, the structure and morphology of the resulting HACC-rGO have been characterized by infrared spectroscopy, X-ray diffraction (XRD) and field emission scanning electron microscopy (FE-SEM) [29]. As shown in Figure 1b, the flake size of the resulting HACC-rGO is approximately 10 μm. In Figure 1c, the peak at 1350 cm^−1^ (usually called the D-band) represents the disordered carbon atoms and structure defects. The peak at 1580 cm^−1^ (usually called the G-band) suggests the presence of crystalline graphitic carbon. The intensity ratio of the D to G-band can be employed to evaluate the structure disorder of carbon materials [30–31]. Thus, the Raman spectroscopy analysis of HACC-rGO suggests that there exist some structural defects in HACC-rGO. Particularly, the resulting HACC-rGO disperses well in aqueous solutions, and it also exhibits good dispersion stability in the solution. Figure 1d shows that HACC-rGO can be well-dispersed in the chitosan solution to form a homogeneous solution, and the mixed solution remains stable even after 24 h. On the contrary, Figure 1e shows that the unmodified rGO does not disperse well in the chitosan solution, and it also has poor dispersion stability in the solution. Thus, HACC-rGO can be well-dispersed in the chitosan solution to form a stable deposition solution, which is extremely beneficial to its further electrodeposition.

**Figure 2:**
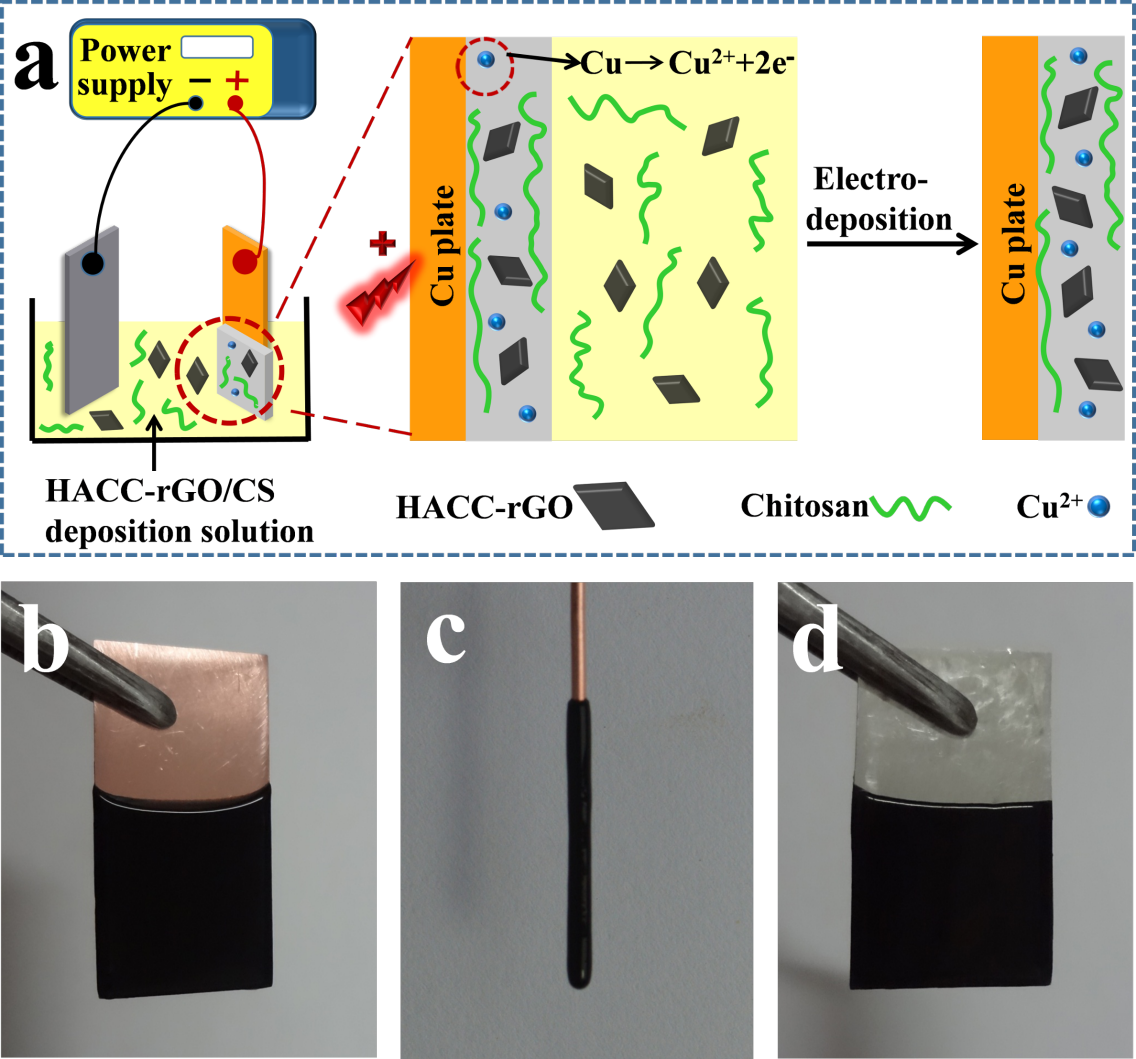
(a) Schematic illustration of the electrodeposition of HACC-rGO with chitosan on the copper electrode. (b) Image of the deposited HACC-rGO/CS film on the copper plate. (c) Image of the deposited HACC-rGO/CS film on the copper wire. (d) Image of the deposited HACC-rGO/CS film on the silver plate.

Recently our group has developed a coordination electrodeposition method for chitosan [13], which can be potentially used in biomedical devices and surface coatings. However, the feature of the coordination deposition method to assemble nanoparticles has not been explored. In this work, the electrodeposition of HACC-rGO with chitosan, based on the coordination deposition method, has been investigated. The schematic of the electrodeposition of HACC-rGO with chitosan on a copper electrode is illustrated in Figure 2a. In the electrodeposition, a copper plate was used as the anodic electrode and a platinum foil was used as the cathodic electrode, and the electrodes were partially immersed into the deposition solution (the mixed solution of HACC-rGO and chitosan), then a voltage of 1.2 V was applied using a DC power supply. During the electrodeposition, the copper plate can be electrochemically oxidized, which then produces Cu^2+^ ions on the surface of the copper plate. Chitosan possesses the capability to coordinate to transition metal ions (e.g., Cu^2+^ and Ag^+^ ions). This coordination capability is related to the existence of amino groups in chitosan. Consequently, chitosan molecules close to the copper plate are able to coordinate to these Cu^2+^ ions to form a stable deposited film on the surface. At the same time, the HACC-rGO in the deposition solution can be codeposited with chitosan and introduced into the deposited film. Finally, the deposited film (named as HACC-rGO/CS film) is generated on the copper plate based on the coordination electrodeposition method.

Figure 2b shows that the homogeneous, deposited HACC-rGO/CS film is generated on the copper plate by electrodeposition, indicating that the electrodeposition of HACC-rGO with chitosan can be accomplished through the above-mentioned method. In addition, the black coloration of the deposited film suggests the presence of HACC-rGO in the film. As shown in Figure 2c, the HACC-rGO/CS film can also be deposited on the copper wire using the same deposition method. In another experiment, a silver plate was used as the anode to carry out the electrodeposition of HACC-rGO with chitosan. It can be seen from Figure 2d that a homogeneous, deposited film is also formed on the silver plate. Consequently, these results indicate that using the aforementioned electrodeposition method we can achieve the codeposition of HACC-rGO with chitosan and build the deposited HACC-rGO/CS films on the copper or silver electrodes/substrates.

### Properties of deposited HACC-rGO/CS films

**Figure 3:**
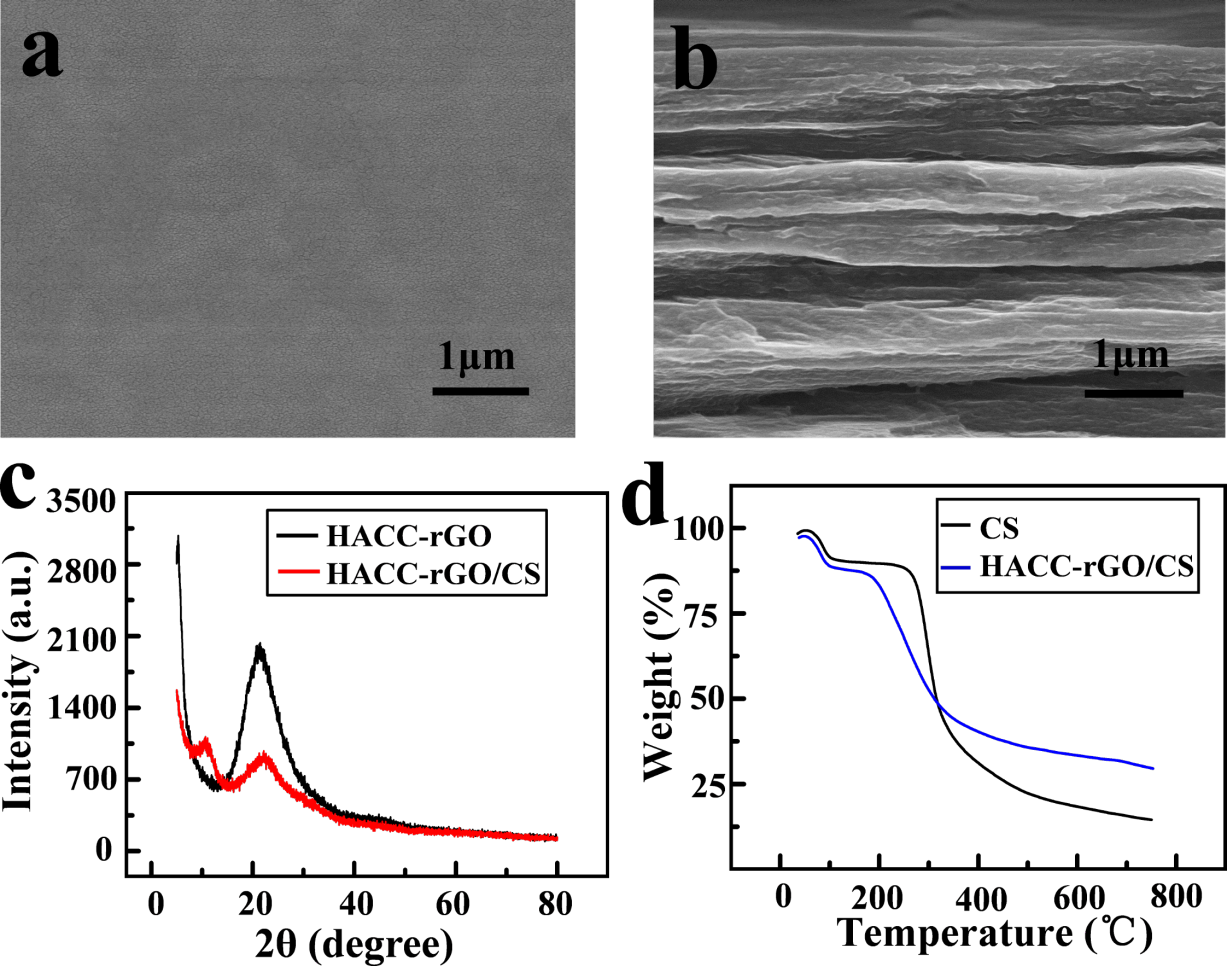
(a) SEM image of the deposited HACC-rGO/CS film surface. (b) SEM image of the cross-section of the HACC-rGO/CS film. (c) XRD patterns of the HACC-rGO powder and the HACC-rGO/CS film. (d) Thermogravimetric analysis of the deposited HACC-rGO/CS film and the control chitosan.

The surface and the cross-section morphology of the deposited HACC-rGO/CS film were observed by SEM. In Figure 3a, the SEM image shows that the surface of the deposited HACC-rGO/CS film is relatively smooth. In Figure 3b, the cross-section morphology of the film shows a layered structure. Similarly, Wu et al. fabricated rGO/poly(vinyl alcohol) composite films, and they also found that the cross-section of composite films exhibit a gradient, layered structure [32]. Figure 3c shows XRD patterns of the HACC-rGO powder and the deposited HACC-rGO/CS films, respectively. The diffraction peak of the HACC-rGO powder appears at 21.4°. In contrast, Maddinedi et al. synthesized rGO by a green, facile method, and the resulting rGO exhibited a diffraction peak at 21.8°, which is similar to the peak of the HACC-rGO [33]. On the other hand, the XRD pattern of the HACC-rGO/CS film shows a diffraction peak at 22.1°, which is attributed to the HACC-rGO in the deposited film. Additionally, the HACC-rGO/CS film presents another diffraction peak at 10.8°, which corresponds to the diffraction peak of chitosan [34–35]. The thermogravimetric analysis (TGA) curves in Figure 3d show that there is a continuous weight loss for the two samples below 600 °C. However, the residues of the HACC-rGO/CS film are obviously higher than that of the chitosan film at 750 °C, which was related to the existence of HACC-rGO. According to the previously described method [36], the weight fraction of rGO is calculated to be 12% in the deposited HACC-rGO/CS film based on the difference between the residues of two samples at 750 °C.

**Figure 4:**
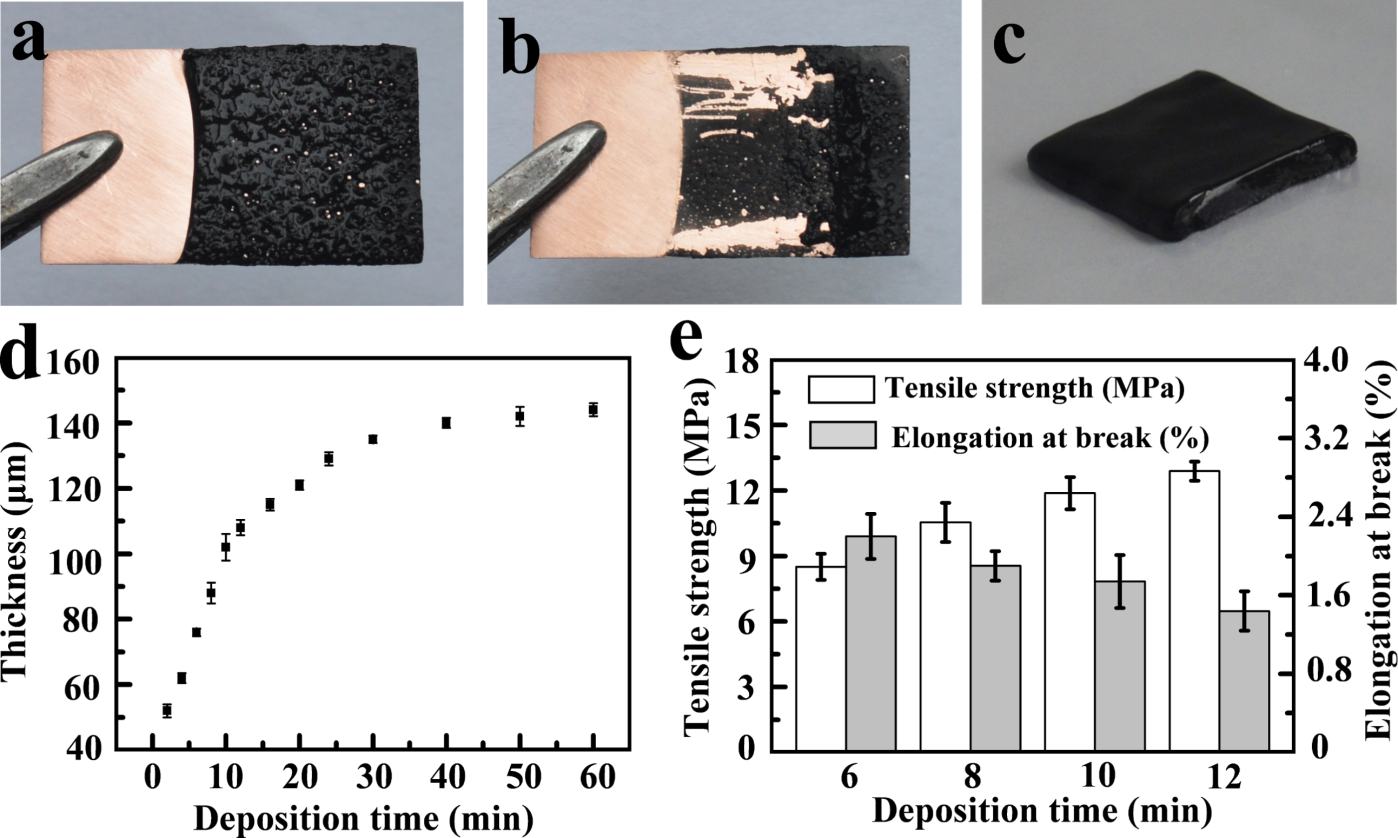
Images of the cathodically electrodeposited film of HACC-rGO and chitosan on the copper plate (a) and peeled from the copper plate (b). (c) Image of the deposited HACC-rGO/CS film peeled from the copper plate. (d) Thickness of the deposited HACC-rGO/CS films at different electrodeposition times. (e) Tensile strength and elongation at break of the HACC-rGO/CS film with different deposition times (6, 8, 10, and 12 min).

It has been reported that chitosan is the first biopolymer to be electrodeposited based on a cathodic neutralization mechanism which is now used in many fields. However, during the cathodic electrodeposition, H_2_ bubbles may be introduced in the deposited chitosan film due to the electrochemical reactions on the cathode, which can produce surface defects that will affect applications of the deposited film. In contrast, we carried out the cathodic electrodeposition of HACC-rGO with chitosan on a copper plate. It is shown in Figure 4a that a black film is deposited on the copper plate after the cathodic electrodeposition. Obviously, the film has a rough surface with many surface defects because of the H_2_ bubbles generated by the cathodic reactions. Moreover, Figure 4b shows that the cathodically deposited film cannot be peeled from the copper plate. On the other hand, the HACC-rGO/CS film fabricated by coordination electrodeposition method is homogeneous, and it can be completely peeled from the copper plate as shown in Figure 4c. Concurrently, the coordination electrodeposition can produce much thicker HACC-rGO/CS films (approximately 2.0 mm, electrodeposition at 1.2 V for 12 min) compared with the cathodic electrodeposition. Thus, the electrodeposition method in this work enables a controllable and novel means to prepare rGO/chitosan films that can be detached from the electrode and used as independent nanocomposite films. Furthermore, we measured the thickness of the dried HACC-rGO/CS film at different deposition times. In Figure 4d the thickness of the HACC-rGO/CS films is shown to gradually increase with the deposition time less than 40 min, suggesting that the thickness of the film can be controlled by external electrical signals. When the deposition time exceeds 40 min, the thickness of the films remains almost unchanged (the maximum thickness of the film is approximately 140 μm). As shown in Figure 4e, the tensile strength of the deposited HACC-rGO/CS films increases with the deposition time (8.5 MPa for 6 min, 10.5 MPa for 8 min, 11.9 MPa for 10 min, and 12.9 MPa for 12 min, respectively). However, the elongation at break of the deposited films decreases slightly with the deposition time.

From the performance analysis described above, it is found that the HACC-rGO/CS film generated by the coordination deposition method has a homogeneous surface, which is beneficial to subsequent applications of the deposited film. In contrast, the cathodically deposited graphene films have a rough surface with many surface defects [20,37]. On the other hand, the HACC-rGO/CS film in this work is able to be completely peeled from the electrodes and used independently. However, the electrodeposited graphene films reported elsewhere were not detached from the electrodes [19,38].

**Figure 5:**
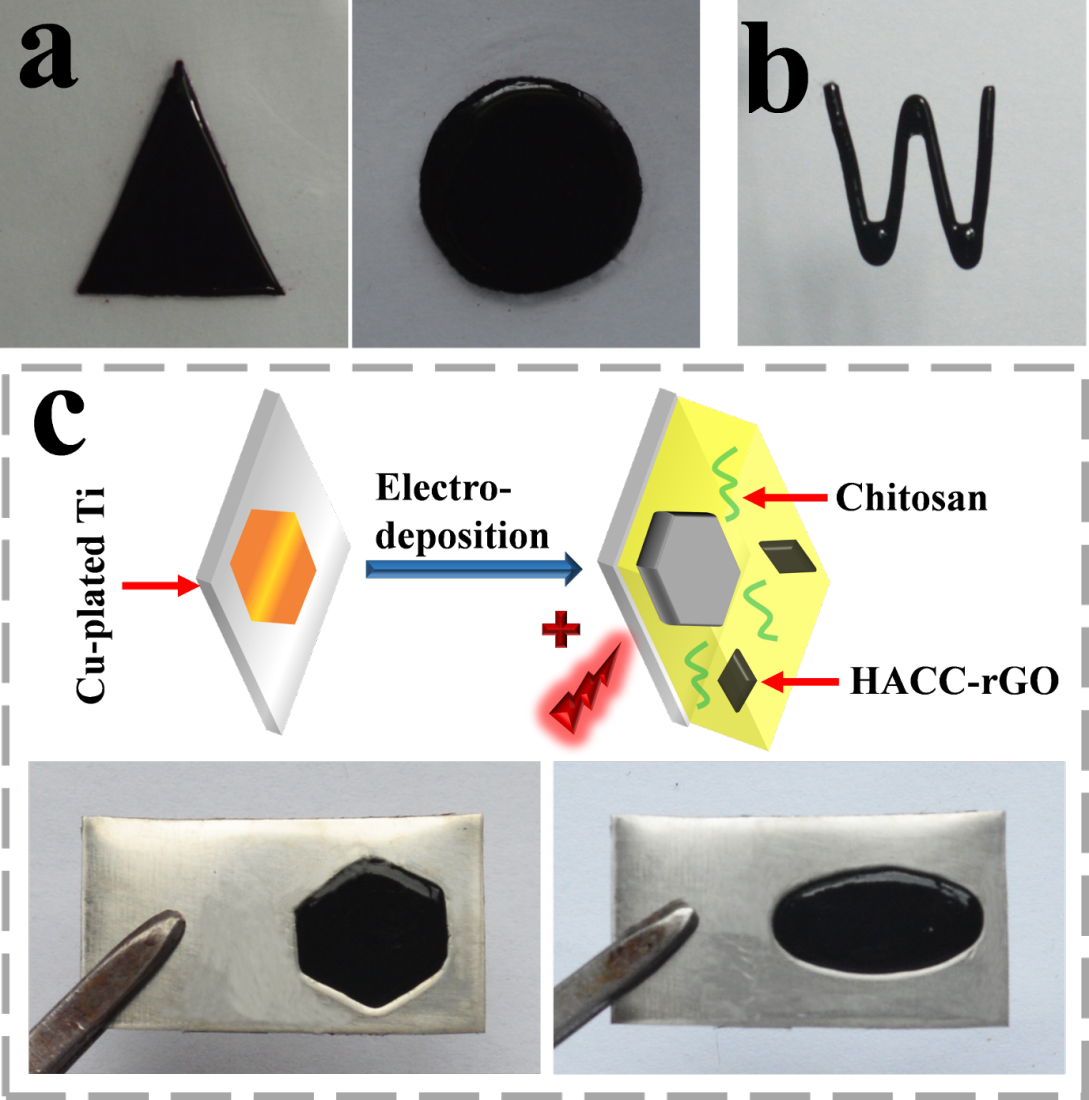
(a) Photographs of the HACC-rGO/CS films (peeled from the copper plates) with various shapes. (b) Photograph of the HACC-rGO/CS film on the copper wire with a “W” shape. (c) Schematic illustration for fabricating the HACC-rGO/CS film with desired shapes on the copper-plated titanium plate (Cu-plated Ti), and photographs of the resulting films.

Subsequently, the deposited HACC-rGO/CS films with various shapes were fabricated by different methods. We initially employed copper plates with specific shapes to prepare the HACC-rGO/CS films of the specific shapes. As shown in Figure 5a, the homogeneous HACC-rGO/CS films with different shapes are created using the aforementioned method. These films can be completely peeled from the copper plates, which is beneficial for their further applications as independent nanocomposite films. Figure 5b reveals that the HACC-rGO/CS film can also be created on the copper wire with a “W” shape, suggesting that we can conveniently build rGO/chitosan nanocomposite coatings on the substrates with special shapes, which provides potential applications in surface coatings and electronic devices. In another method, we fabricated the deposited HACC-rGO/CS films with desired shapes on titanium plates which were electroplated with copper in the desired region and shape. Figure 5c shows that the HACC-rGO/CS films with regular shapes can be achieved on the titanium plates. The HACC-rGO/CS films are only deposited on the titanium plates at the copper-plated regions, whereas they cannot deposit on the region without the electroplated copper. Consequently, the above results both reveal that the electrodeposition method in this work provides a controllable means to fabricate the rGO nanocomposite films in different shapes.

### Electrodeposited HACC-rGO/CS films for electrochemical detection

Graphene has drawn extensive interest in sensor applications due to its favorable physical and electrochemical properties. In addition, there are great advantages in the use of graphene and graphene-based nanocomposites for the fabrication of electrochemical sensors with enhanced detection performance [39]. It has been reported that graphene-based materials can be enlisted for the electrochemical detection of glucose, NH_3_ gas, NO_2_ gas, ethanol and acetone [40–43]. Besides, graphene-based sensors have been used to detect phenolic compounds which are aromatic pollutants to the environment and human health [44–45]. These graphene-based sensors exhibit the high detection performance for phenolic compounds owing to the special advantages of graphene such as its excellent electronic properties, large surface area, and high adsorption capability for phenolic compounds [44–45].

**Figure 6:**
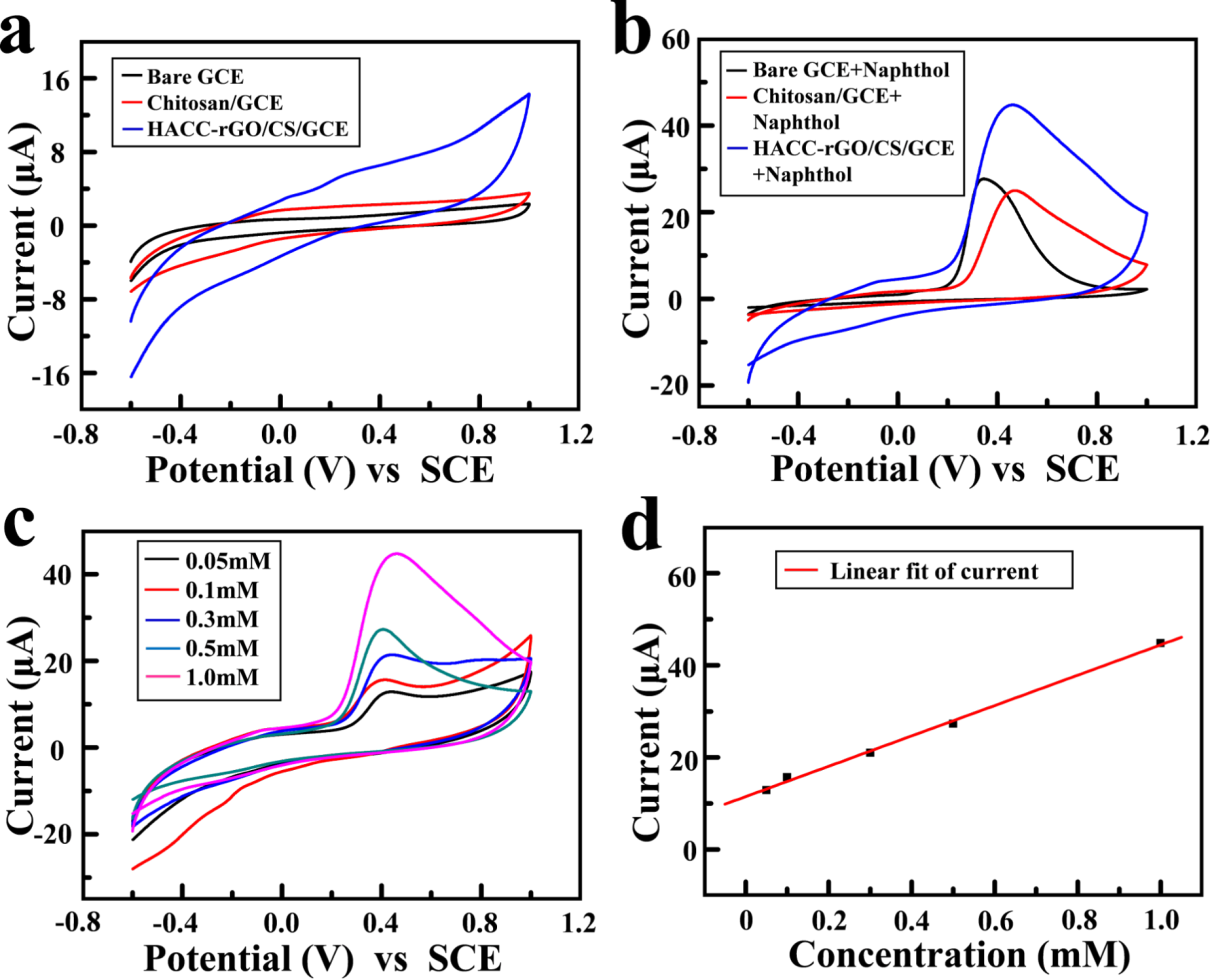
CV curves of the bare GCE, the GCE with the deposited chitosan film (chitosan/GCE), and the GCE with the deposited HACC-rGO/CS film (HACC-rGO/CS/GCE) in the absence 1-naphthol (a) and in the presence of 1.0 mM 1-naphthol (b), scan rate 0.1 V s^−1^. (c) CVs of the GCE with the deposited HACC-rGO/CS film in the presence of 1-naphthol with different concentrations (0.05, 0.1, 0.3, 0.5 and 1.0 mM), scan rate 0.1 V s^−1^. (d) Correlation between the current and the concentration of 1-naphthol.

As a proof-of-concept experiment, the electrochemical detection capability of the deposited HACC-rGO/CS film on a glassy carbon electrode (GCE) to detect 1-naphthol (as a model analyte) was explored. The electrochemical measurements were carried out using a three-electrode system with the GCE as the working electrode, a saturated calomel electrode as the reference electrode, and a platinum wire as the counter electrode. Figure 6a and Figure 6b show the cyclic voltammograms (CVs) of a bare GCE, a GCE with the deposited chitosan film (without HACC-rGO) and the GCE with the deposited HACC-rGO/CS film in the absence and in the presence of 1.0 mM 1-naphthol. No obvious peak is observed in the CV curve of the bare GCE in the absence of 1-naphthol, as well as the CVs of the GCE with the deposited chitosan film and the GCE with the deposited HACC-rGO/CS film in the absence of 1-naphthol. In contrast, there are obvious peaks near the potential of 0.4 V in the CVs of the three electrodes in the presence of 1.0 mM 1-naphthol, which could be attributed to the electrochemical oxidation of 1-naphthol [45]. In particular, the CV of the GCE with the deposited HACC-rGO/CS film presents the largest peak current, suggesting that the electrodeposition of HACC-rGO on the electrode can enhance the electrochemical detection capability for 1-naphthol. Furthermore, the CVs in Figure 6c confirm that there are peak currents near 0.4 V at different 1-naphthol concentrations, and the peak currents systematically increase with the concentration of 1-naphthol. Additionally, the plot in Figure 6d further reveals that there is a correlation between the current and the concentration of 1-naphthol.

**Figure 7:**
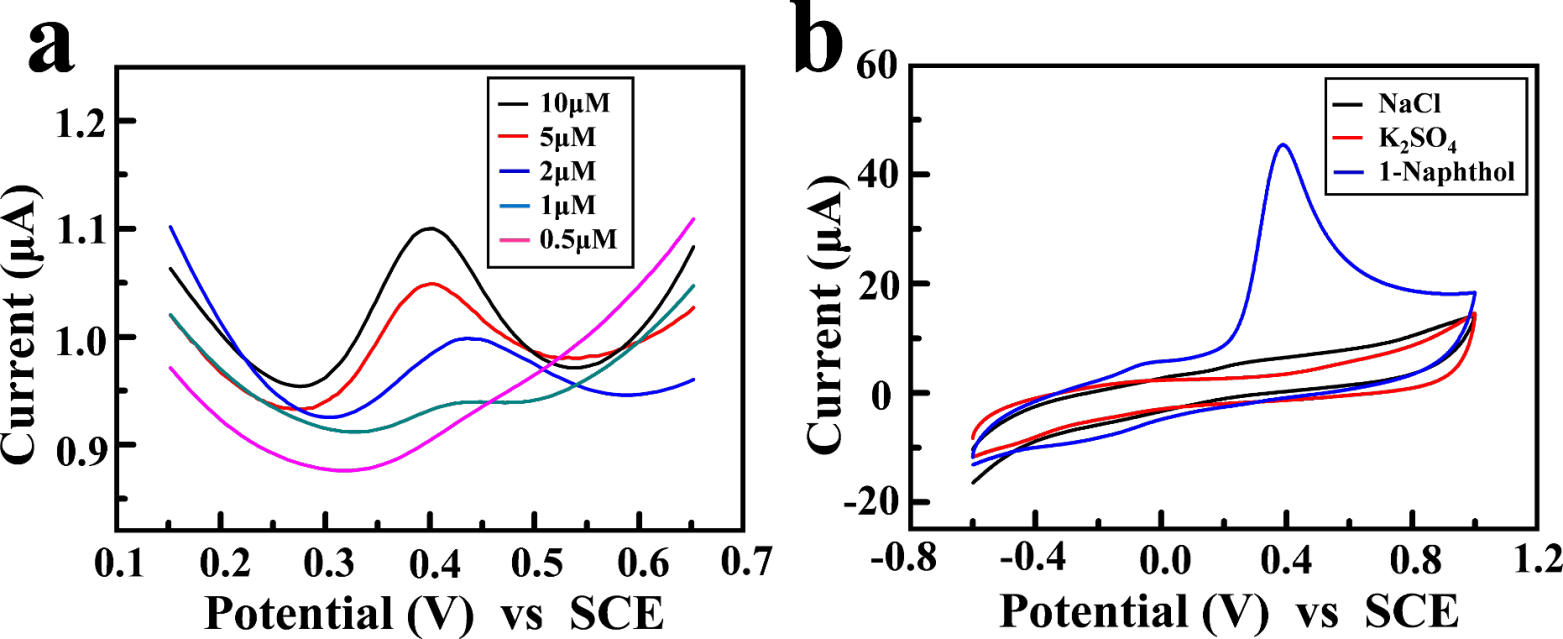
(a) DPV of the GCE with the deposited HACC-rGO/CS film in the presence of 1-naphthol with different concentrations (0.5, 1, 2, 5 and 10 μM). (b) CV curves of the GCE with the deposited HACC-rGO/CS film in the presence of 1.0 mM NaCl, 1.0 mM K_2_SO_4_, and 1.0 mM 1-naphthol. Scan rate 0.1 V s^−1^.

Figure 7a shows the differential pulse voltammetry (DPV) of the GCE with the deposited HACC-rGO/CS film in the presence of 1-naphthol with different concentrations. The peak current for 1-naphthol increases with the increase of 1-naphthol concentration from 0.5 to 10 μM. It is shown that the detection limit for 1-naphthol is approximately 0.5 μM. In contrast, Wang et al. reported that GCE modified with carbon nanotube networks joined by Pt nanoparticles has the detection limit of 0.5 μM for 1-naphthol detection, which is similar to that of our work [46]. Additionally, Figure 7b shows that there is an obvious peak current in the CV for 1.0 mM 1-naphthol, whereas there is no obvious peak current in the CVs for 1.0 mM NaCl and 1.0 mM K_2_SO_4_, indicating that the GCE with the deposited HACC-rGO/CS film has an anti-interference capability towards 1-naphthol. Therefore, using the electrodeposition method in this work, we can straightforwardly and conveniently build the deposited rGO/chitosan nanocomposite film on the electrode for electrochemical detection.

## Conclusion

In this work, we developed an electrodeposition method for the direct deposition of HACC-rGO with chitosan, based on the coordination deposition method. Using this method, we can conveniently build homogeneous HACC-rGO/chitosan films on copper or silver electrodes or substrates. In addition, the deposited films can be completely peeled from the electrodes, which provides a controllable way to prepare independent rGO/chitosan nanocomposite films. By making use of the electrodeposition, we can conveniently and controllably build rGO/chitosan nanocomposite coatings or films of various shapes. This method was used to deposit rGO/chitosan films on an electrode for the electrochemical detection of 1-naphthol. Thus, the electrodeposition method for reduced graphene oxide in this work provides promising applications in nanocomposite films, functional coatings, and biodevices.

## Experimental

### Chemicals and materials

Natural graphite powder (325 mesh), chitosan (90% deacetylation degree), concentrated sulfuric acid, hydrochloric acid, sodium nitrate, potassium permanganate, hydrogen peroxide, hydrazine hydrate, acetic acid, sodium hydrate, and 1-naphthol were purchased from Sinopharm Chemical Reagent Co., Ltd., China. 2-Hydroxypropyltrimethylammonium chloride chitosan was purchased from Nantong Green Biological Co., Ltd., China. The copper plates, silver plates, titanium plates, platinum foil, glassy carbon electrode and other chemicals were obtained from various commercial sources in China. All chemicals were of analytical grade and were not purified before use.

### Preparation of modified rGO

HACC-rGO was prepared using a previously reported method [29]. Briefly, 100 mg of GO was dispersed in 30 mL of 0.05 M NaOH solution by ultrasonication, and the GO dispersion was added in 100 mL of HACC aqueous solution (0.1% w/v) to generate HACC-GO. Then the pH of the solution was adjusted to 10 with 0.1 M NaOH. Subsequently, HACC-GO was reduced to HACC-rGO by adding 0.4 g of hydrazine hydrate to the solution, and reacting at room temperature for 20 min, then reacting at 80 °C for 2 h. Finally, the resulting products were filtered, washed with distilled water, and vacuum dried to obtain HACC-rGO.

### Preparation of HACC-rGO and chitosan deposition solution

The HACC-rGO dispersion (0.6% w/v) was prepared by adding the above described HACC-rGO powder in distilled water, followed by ultrasonication for 20 min. The chitosan solution (2.0% w/v) was prepared by dissolving the chitosan powder in acetic acid (0.25% v/v), and adjusting pH to 5.5, then filtering to remove the undissolved particles [13]. Next, the deposition solution (0.3% w/v HACC-rGO and 1.0% w/v chitosan solution) was prepared by mixing equal volumes of HACC-rGO dispersion and chitosan solution. In contrast, the mixture of unmodified rGO (0.3% w/v) and chitosan (1.0% w/v) was prepared by mixing equal volumes of unmodified rGO dispersion (0.6% w/v) and chitosan solution (2.0% w/v).

### Electrodeposition of HACC-rGO with chitosan

The electrodeposition of HACC-rGO with chitosan was carried out according to the previously reported coordination deposition method [13]. A copper plate (or a copper wire) was used as the anodic electrode, and a platinum foil served as the cathodic electrode. Before use, both electrodes were carefully polished, and then ultrasonically washed in acetone, ethanol and distilled water for 10 min each. Then, the electrodeposition was conducted using a programmable DC power supply (IT6123, TW). Both the cathode and the anode were partially immersed into the deposition solution, and then a DC voltage of 1.2 V was applied. After a given electrodeposition time (e.g., 9 min), the anode was disconnected from the power supply, and removed from the deposition solution, then rinsed with distilled water. Finally, the deposited HACC-rGO/CS film on the copper electrode was obtained.

Also, the electrodeposition of HACC-rGO and chitosan was performed by using a silver plate as the anode and a platinum foil as the cathode. After electrodeposition, the deposited film on the silver plate was rinsed with distilled water. In contrast, the cathodic electrodeposition of HACC-rGO and chitosan was carried out on the electrode. In brief, a copper plate was used as the cathode and a platinum foil was used as the anode, then the cathode and the anode were partially immersed into the deposition solution to perform the cathodic electrodeposition using a programmable DC power supply (2.5 V, 9 min). Finally, the cathodically deposited film was then achieved on the copper plate.

### Fabrication of deposited HACC-rGO/CS films of various shapes

The deposited HACC-rGO/CS films with desired shapes were initially fabricated by employing electrodes having the desired, targeted shapes. Briefly, the copper electrodes were formed into the desired shapes and carefully polished and ultrasonically washed before use. Then, these electrodes were electrodeposited in the aforementioned deposition solution to obtain the HACC-rGO/CS films with desired shapes on the electrodes or peeled from the electrodes. Similarly, the copper wires were made into different shapes, and then electrodeposited in the deposition solution to achieve the deposited HACC-rGO/CS films with different shapes.

Furthermore, the deposited HACC-rGO/CS films with different shapes were fabricated on titanium plates. Briefly, a titanium plate was coated with an alcohol-soluble ink (mainly composed of polyvinyl butyral resin) at the desired region as the protective coating, and then it was electroplated with copper at the desired region by the previously reported method [13]. Subsequently, the copper-plated titanium plate was used as the anode and a platinum foil was used as the cathode. The electrodeposition was then performed in the deposition solution according to the above-mentioned method, and finally, the deposited HACC-rGO/CS film with the desired shape was achieved on the titanium plate after the electrodeposition.

### Electrochemical detection experiments

To start with, a glassy carbon electrode with the deposited HACC-rGO/CS film was fabricated by the following method. The GCE was used as the cathode and a copper plate was used as the anode, then the electrodes were immersed in 0.1 M CuSO_4_ solution to electroplate copper on the GCE at a DC voltage of 1.5 V. Next, the copper-plated GCE was used as the anode and a platinum foil was used as the cathode, and the electrodeposition was performed in the 0.3% w/v HACC-rGO and 1.0% w/v chitosan solution for a time long enough to completely consume the electroplated copper. After electrodeposition, the GCE with the deposited HACC-rGO/CS film was taken out from the deposition solution and rinsed with distilled water. Subsequently, the electrochemical detection experiments for detecting 1-naphthol were carried out on a CHI 618E electrochemical analyzer (CH Instruments, Chenhua Co., Shanghai, China) using a three-electrode system. The GCE with the deposited HACC-rGO/CS film as the working electrode, a saturated calomel electrode (SCE) was used as the reference electrode, and a platinum wire served as the counter electrode.

### Characterization

The X-ray diffraction pattern of the HACC-rGO powder and the deposited HACC-rGO/CS film were performed on an X-ray diffractometer (D/MX-IIIA, Rigaku, JP). The flake size of the HACC-rGO material was observed using a field emission scanning electron microscope (JSM-5610LV, JEOL Ltd., Japan). The Raman spectrum of the HACC-rGO sample was measured using a Raman spectrometer (INVIA, Renishaw, UK) with an excitation wavelength of 633 nm. The surface and the cross-section morphology of the deposited HACC-rGO/CS film were observed by a field emission scanning electron microscope (JSM-5610LV, JEOL Ltd., JP). The tensile strength and elongation at break of the deposited HACC-rGO/CS film were tested by a universal testing machine (CMT6503, Shenzhen SANS Test Machine Co., China). The area of the film for the test was 30 × 10 mm, and the cross-head speed was 10 mm/min. Each final measurement was obtained from the average value of five samples. The thermogravimetric analysis of the sample was performed using a thermogravimetric analyzer (STA449c/3/G, Netzsch, Germany) with a heating rate of 10 °C/min under nitrogen atmosphere.
